# Inference and Forecast of the Current West African Ebola Outbreak in Guinea, Sierra Leone and Liberia

**DOI:** 10.1371/currents.outbreaks.3408774290b1a0f2dd7cae877c8b8ff6

**Published:** 2014-10-31

**Authors:** Jeffrey Shaman, Wan Yang, Sasikiran Kandula

**Affiliations:** Environmental Health Sciences, Columbia University, New York, New York, USA; Environmental Health Sciences, Columbia University, New York, New York, USA; Environmental Health Sciences, Columbia University, New York, New York, USA

## Abstract

The current West African Ebola outbreak poses an unprecedented public health challenge for the world at large. The response of the global community to the epidemic, including deployment of nurses, doctors, epidemiologists, beds, supplies and security, is shaped by our understanding of the spatial-temporal extent and progression of the disease. Ongoing evaluation of the epidemiological characteristics and future course of the Ebola outbreak is needed to stay abreast of any changes to its transmission dynamics, as well as the success or failure of intervention efforts. Here we use observations, dynamic modeling and Bayesian inference to generate simulations and weekly forecasts of the outbreaks in Guinea, Liberia and Sierra Leone. Estimates of key epidemiological characteristics over time indicate continued epidemic growth in West Africa, though there is some evidence of slowing growth in Liberia. 6-week forecasts over successive weeks corroborate these findings; forecasts projecting no future change in intervention efficacy have been more accurate for Guinea and Sierra Leone, but have overestimated incidence and mortality for Liberia.

## Introduction

The current West African Ebola outbreak is unprecedented in magnitude and duration^1^^,^ 2. How the outbreak will progress in the three most afflicted countries, i.e., Guinea, Liberia, and Sierra Leone, whether the outbreak can be contained geographically, and the extent and optimal implementation of resources needed to extinguish the outbreak remain uncertain. These uncertainties present enormous challenges for the medical and public health communities, and the world at large.

Ebola containment and control efforts are, in part, directed by our understanding of the state and projected spread of the outbreak; however, observations of the current West African Ebola outbreak are fragmented and incomplete. In the absence of comprehensive observational data, modeling and computation analysis can be used to estimate a more complete picture of transmission dynamics. In the present situation, continued estimation of the evolving epidemiological characteristics of Ebola in West Africa is needed to track and respond to the outbreak as it changes through time. In addition, short-term forecasts of the progression of the outbreak can be used to help guide the allocation of resources and personnel. These forecasts, made over successive weeks can also be compared with observed outcomes once they have come to pass, to provide additional information on whether outbreak dynamics have shifted.

Recent advances in infectious disease modeling, in particular the development of systems combining mathematical modeling and Bayesian inference, have enabled estimation of key epidemiological characteristics associated with historical disease outbreaks^3^ , including partially observed systems, as well as the accurate real-time forecast of some disease systems, such as influenza^4^^,^ 5 . The Bayesian inference, or data assimilation, methods employed partially compensate for both observational error and model misspecification, and, by doing so, provide a better estimate of system behavior than analysis using the data or model alone.

The question pursued here is whether these techniques can be sensibly applied to the current Ebola outbreak given the large uncertainties associated with the disease, e.g., the under-reporting of the current outbreak^2^ , and the difficulty of modeling a disease system with important, under-resolved social and spatial characteristics. Here we use World Health Organization (WHO) observations, dynamic modeling and Bayesian inference to estimate key epidemiological characteristics and forecast future case and mortality levels for the current West African Ebola outbreak. Forecasts made in prior weeks are compared with subsequently observed incidence and mortality levels and used to assess model performance and the current outlook for the outbreak. The intent is to provide bounds on the epidemiology of the current outbreak and its possible future course.

## Methods

**Incidence and Mortality Data**

Cumulative Ebola incidence and mortality data were derived from WHO’s Disease Outbreak News and situation reports for Guinea, Liberia and Sierra Leone^6^ . For this study, cumulative incidence included all confirmed, probable and suspect cases at the date of reporting. Cumulative deaths were derived similarly. As the WHO reports were issued irregularly in time, the incidence and mortality data were interpolated to weekly intervals to allow generation of regular, weekly parameter estimates and forecasts.

**SEIRX Model**

Ebola is a disease with complicated transmission dynamics. While the suspected mode of transmission has been generally identified—contact with bodily fluids of infected hosts^7^ —the specific rates of spillover from zoonotic reservoirs and chains of transmission within the human population have been poorly documented for the current West African outbreak. In addition, in the current context, response and mitigation efforts are being implemented non-uniformly and with varying efficacy in both space and time. Consequently, it is difficult to specify an appropriate model structure to represent these complex, changing dynamics. Indeed, it is not clear whether to work with a compartmental, meta-population or agent-based approach. Our understanding of the outbreak suggests a more stylized, complex approach with zoonotic spillover and spatially resolved simulation of the spread of the virus is warranted; however, the limited data available support using a more parsimonious model structure.

Model parsimony does not preclude effective, meaningful inference and forecast. As an analogy, the contact patterns and even dominant mode of transmission for influenza remain largely unknown^8^ ; however, skillful predictions of influenza outbreak characteristics can still be generated using very simple compartmental models, imperfect observations and data assimilation methods^5^^,^ 9 ^,^ 10 . Here we apply those same model-data assimilation methods to the current Ebola outbreak. We use simple spatially-unresolved, perfectly-mixed compartmental model forms, but attempt to capture implicitly some of the specific characteristics associated with Ebola transmission, including the spatial and temporal heterogeneity of Ebola transmissibility, e.g. the changes in transmission dynamics due to behavioral changes among the population. As for influenza inference and forecast, these spatial characteristics are not represented explicitly in the model form, but this model misspecification is partially compensated for by the applied data assimilation methods.

Prior modeling studies of Ebola have used expanded versions of a susceptible-exposed-infectious-recovered (SEIR) model, in which additional compartments are employed to describe the Ebola transmission cycle more completely^11^^,^ 12 ^,^ 13 . For this effort, we add a compartment, *X,* for the deceased population to allow assimilation of mortality and case fatality rate data in addition to incidence. The model is described by the following equations:

\begin{equation*}\frac{dS}{dt}=-\frac{\beta(t)IS}{N}-\alpha\end{equation*} [1]

\begin{equation*}\frac{dE}{dt}=\frac{\beta(t)IS}{N}-\frac{E}{Z}+\alpha\end{equation*} [2]

\begin{equation*}\frac{dI}{dt}=\frac{E}{Z}-\frac{(1-\eta)I}{D}-\frac{\eta{I}}{M}\end{equation*} [3]

\begin{equation*}\frac{dR}{dt}=\frac{(1-\eta)I}{D}\end{equation*} [4]

\begin{equation*}\frac{dX}{dt}=\frac{\eta{I}}{M}\end{equation*} [5]

where *S* is the number of susceptible people in the population, *t* is time in years, *N* is the population size, *I* is the number of infectious people, \begin{equation*}\small{\beta{(t)}}\end{equation*} is the transmission rate exerted by infectious persons at time *t*, *α* is the rate of Ebola import (either from outside populations or zoonotic sources) into the model domain, *E *is the number of exposed people, *Z* is the average time before an exposed person becomes infectious (i.e. the incubation period), *D* is the mean infectious period, *M *is the average time from symptom onset to death, *X *is the**number deceased, and *η* is the case fatality rate.

The transmission rate exerted by the live infectious persons, \begin{equation*}\small{\beta{(t)}}\end{equation*}, is a stochastic variable defined as:

\begin{equation*}\beta{(t)}=(R_{0Mean}+\kappa{R_{0Amp}})/D\end{equation*} [6]

where \begin{equation*}\small{R_{0Mean}}\end{equation*} is the mean basic reproductive number, i.e. the number of secondary infections the average infectious person would produce in a fully susceptible population, \begin{equation*}\small{R_{0Amp}}\end{equation*} is the amplitude with which the daily reproductive number, \begin{equation*}\small{R_0(t)= R_{0Mean}+\kappa{ R_{0Amp}}}\end{equation*}, varies around \begin{equation*}\small{R_{0Mean}}\end{equation*}, and *κ* is a number drawn randomly from the uniform distribution *U*[-0.5, 0.5].

The variations of *R*_0_(*t*) are devised to represent changes in the force of transmission, the magnitude of which could differ in both space and time, depending on factors related to the spread of Ebola, e.g., changes in social behavior within the local community, intervention practices, and funeral practices. As such, the stochastic variable *R*_0_(*t*) implicitly recognizes that within each country there is considerable heterogeneity of Ebola transmission, even though we simulate the Ebola outbreaks in Guinea, Sierra Leone and Liberia using a perfectly-mixed SEIRX model. For example, an increase in *R*_0_(*t*), i.e.\begin{equation*}\small{R_0(t)\rightarrow R_{0Mean}+0.5R_{0Amp}}\end{equation*}, could reflect events such as when Ebola appears in a new district or village, and a decrease in *R*_0_(*t*), i.e. \begin{equation*}\small{R_0(t)\rightarrow{R_{0Mean}-0.5R_{0Amp}}}\end{equation*}, could mirror successful implementation of intervention and care measures in response to these new emergences. As the time series of these variations are fundamentally unknown, a stochastic formulation for *R*_0_(*t*) is applied; the values of *R*_0Mean_ and *R*_0Amp_, which determine the value of *R*_0_(*t*), are selected through the data assimilation process (described below).

During the early portions of the outbreak, when the distribution and numbers of infection were more limited, new Ebola cases appeared erratically (Figure 1). This circumstance may reflect errors in reporting; however, it also may reflect variations in the force of transmission as the outbreak spread into new regions and intervention and control efforts proved more or less successful. Even when smoothed, the incidence time series remains erratic. Importantly, simulation with this SEIRX framework (Equations 1-5) and the stochastic formulation for *R*_0_(*t*) (Equation 6) can produce an Ebola incidence time series similar to these observations (Figure 1). Without a stochastic component varying *R*_0_(*t*), as in Equation 6, the SEIRX cannot produce a time series like the observed.

**The erratic time series of Ebola incidence data.**
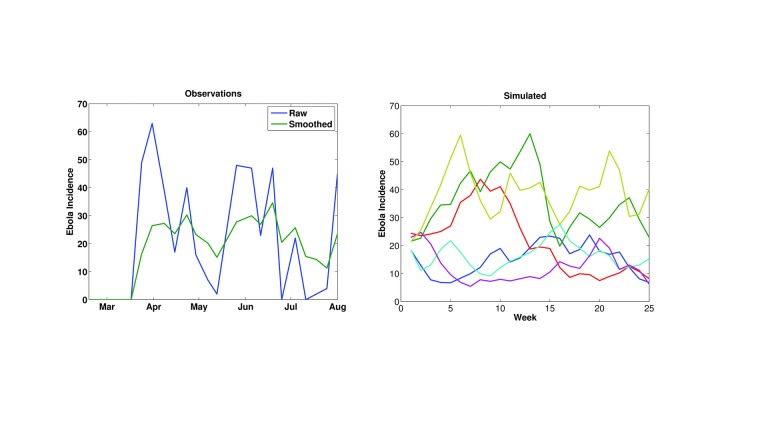
Left) Time series of WHO Ebola incidence in Guinea for mid-February through July 2014. Raw incidence (blue) and 3-week smoothed incidence (green) are shown. Right) Simulated incidence using the SEIRX model (Equations 1-5) and stochastic variation of R0 (Equation 6). 6 different integrations are shown; all generated with the following initial conditions and parameters: N = 200,000 persons; S(0) = 199,950 persons; E(0) = 25 persons; I(0) = 25 persons; η = 0.5; Z = 8 days; D = 10 days; M = 8 days; α = 0.1/day.

Three hundred-member ensemble simulations with the SEIRX model were run with the WHO country-wide observations of weekly cumulative incidence, mortality, and case-fatality-rate, and the ensemble adjustment Kalman filter (EAKF)^14^ . The EAKF algorithm iteratively updates the ensemble simulations of the observed state variables (i.e. incidence) to better align with observations. These updates are determined by halting the ensemble integration at each new observation, computing the Kalman gain using that new observation and the distribution of current model states (the prior), and then using the Kalman gain to calculate a posterior for the observed state variables. The EAKF then uses cross ensemble co-variability to adjust both the unobserved state variables and model parameters. The posterior is then integrated to the next observation and the process is repeated. In so doing, the ensemble simulations are optimized to match observed incidence and mortality levels and estimate other key variable and parameter characteristics needed to better mimic local outbreak dynamics. Additional details on the application of the EAKF to infectious disease models are provided in Shaman and Karspeck^9^.

Note that all SEIRX variables and parameters are adjusted with each weekly observational update. Consequently, even though only the variables and *R*_0_(*t*) parameter are explicitly time varying within the SEIRX model system of equations, in practice, all parameters vary through time as the EAKF makes adjustments. These parameter updates reflect the ongoing optimization or fitting of the model to the observed time series, as well as possible changes in the underlying dynamics of the epidemic (e.g. a change in the average incubation period).

For calculation of the Kalman gain, we tally cumulative incidence within each ensemble simulation. Cumulative incidence is not a state space variable within the SEIRX model; however, this quantity is tallied within each ensemble simulation to enable comparison with the latest observation of this quantity and calculation of the Kalman gain, per EAKF methodology. We have described this use of incidence in prior work^9^ . We chose cumulative incidence over weekly incidence as it yields smoother parameter estimates through time.

Adaptive inflation^15^^,^ 16 was applied following the assimilation of the weekly observations of incidence, mortality and case fatality rates. The inflation was used to counter EAKF’s tendency toward ‘filter divergence', which occurs when the prior ensemble spread becomes spuriously small, causing the system to give too little weight to observations and to diverge from the true trajectory.

**Forecasts**

Following each assimilation of weekly observations, the ensemble simulations were integrated 6 weeks into the future without further modification. These weekly ensemble forecasts were generated using the latest posterior estimates of the model state variables and parameters, and different values of *κ *in Equation 6, representing 3 scenarios: 1) a ‘no change’ forecast simulation with*κ*drawn randomly from the uniform distribution *U*[-0.5, 0.5] each week for each ensemble member, i.e., \begin{equation*}\small{R_0(t)=R_{0Mean}+\kappa{R_{0Amp}}}\end{equation*}; 2) an ‘improved’ forecast simulation made with a fixed *κ *= −0.5 such that \begin{equation*}\small{R_0=R_{0Mean}-0.5R_{0Amp}}\end{equation*} over the entire forecast period; and 3) a ‘degraded’ forecast with a fixed *κ *= 0.5 such that \begin{equation*}\small{R_0=R_{0Mean}+0.5R_{0Amp}}\end{equation*}. The first scenario assumes a continuation of current intervention efficacy. The second scenario depicts more effective outbreak intervention; the third scenario depicts higher future epidemic growth. For both the improved and degraded scenarios, the magnitude of the imposed *R*_0_ change is necessarily speculative though loosely based on the levels needed to generate the early erratic incidence time series (Figure 1).

Each week for each scenario we generated 50 300-member ensemble forecasts, each initiated with a different random draw of initial conditions. By using the model optimized parameters and initial forecast conditions, as well as the two alternate scenarios (improved and degraded), to generate predictions over successive weeks we can begin to assess forecast accuracy.

**Sensitivity Tests Using Synthetic Time Series**

Prior to working with WHO Ebola observations, model-simulated time series of incidence, case fatality rates, and mortality were generated using free simulation of the SEIRX model. The model-generated time series of incidence and mortality, and these synthetic data were used as a set of observations against which the simulation and parameter estimation abilities of the SEIRX-EAKF framework could be tested. Sensitivity tests were performed in which the model structure, population size, and initial parameter ranges were varied. Incidence, mortality and case fatality rates were all well estimated, as were the infection period and time to death parameters (Figure S1). *R*_0Mean_ was also well estimated; however, the incubation period and *R*_0Amp_ showed only limited constraint, and the variable for the number of exposed persons, *E*, was not as well estimated as other model variables.

The weak constraint of *R*_0Amp_ using the synthetic data indicates there is modest ability to estimate the scale of random fluctuations of *R*_0_ directly from the data using our SEIRX-EAKF framework. Indeed, the estimate of *R*_0Amp _remains near the mean of its initially specified range. However, we retain this parameter and Equation 6, as its presence in the model framework improved the estimates of other parameters (not shown). In addition, when applied to actual Ebola data, some differentiation of this parameter was evident among Guinea, Liberia, and Sierra Leone.

**Initial Conditions**

Initial model conditions for ensemble runs were chosen randomly from the following uniform state variable and parameter ranges: *R*_0Mean_ ~ U[0.5, 8]; *R*_0Amp_ ~ U[0, 3]; *Z*, *D *and *M ~ *U[4, 14] or U[4, 21]; *E*(0) and *I*(0) ~ U[0, 50]; *X*(0) ~ U[0, 10]; and *S*(0) ~ U[0.9*N*, *N*], where *N *= 2 million, is the simulated population size for each country. Table 1 lists all state variables and parameters estimated using the EAKF and the uniform ranges from which their initial values were drawn at the beginning of each ensemble simulation.

**Table 1:** SEIRX state variables and parameter estimated during simulation with EAKF, and the range of values from which initial conditions for each ensemble member were randomly selected.

Variable or Parameter	Initial Uniform Range (Minimum, Maximum)
*S*	[0.9*N, N*]
*E*	[0, 50]
*I*	[0, 50]
*R*	[0, 0.1*N*]
X	[0, 10]
*R*_0Mean_	[0.5, 8]
*R*_0Amp_	[0, 3]
*Z*	[4, 14] or [4, 21]
*D*	[4, 14] or [4, 21]
*M*	[4, 14] or [4, 21]
*η*	[0.5, 0.7]

## Results

With each new set of weekly observations of Ebola incidence and mortality, the EAKF adjusts the model ensemble state variables and parameters. As the model is trained on more data, the parameter estimates can converge toward values that are optimal for simulating the unfolding epidemic, given the model structure. Estimates of these parameters, while specific to the model form, data error and biases, as well as the data assimilation method, provide some insight into the epidemiological characteristics of the current Ebola outbreak in West Africa (Figure 2). During August and September, *R*_0_(t) estimates of were between 1 and 2 for Guinea and Sierra Leone, whereas for Liberia they were considerably higher during June through early September but more recently dropped below 2. The most recent estimates of *R*_0Mean_, generated with data through September 28, 2014, are 1.30 (1.06, 1.61; mean and 95% credible interval), 1.69 (1.38, 2.00), and 0.99 (0.85, 1.13), for Guinea, Liberia, and Sierra Leone, respectively. Since late July, some differentiation of *R*_0Amp_ estimates among countries was also apparent: through September 28, 2014 these values were 1.89 (1.33, 2.43), 1.72 (1.29, 2.28), and 1.45 (1.07, 1.9) for Guinea, Liberia, and Sierra Leone.

**Parameter estimates through time for Guinea, Liberia, and Sierra Leone as generated using the ensemble SEIRX-EAKF framework.**
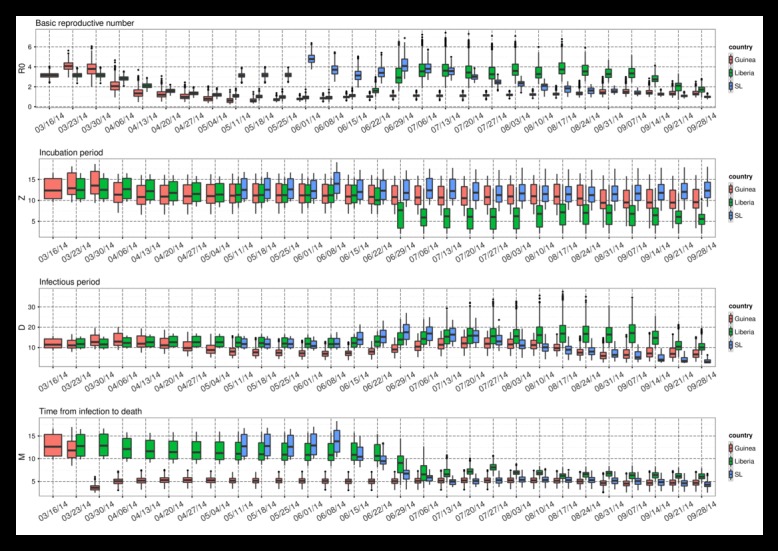
Shown are R0Mean, Z, D, and M. The box and whiskers show the median (black horizontal line), 25th and 75th percentiles (box boundaries), 95% credible interval (whiskers) and outliers (dots).

Estimates of the incubation period, *Z*, the infectious period, *D*, and time from symptom onset to death, *M, *adjusted most precipitously around the end of June, when weekly reported case levels increased substantially. As of September 28, 2014, the incubation period is 10.75 days (7.21, 14.82), 5.87 days (4.05, 8.40), and 12.61 days (9.11, 16.17) and the infectious period is 7.08 days (4.26, 10.25), 9.99 days (7.48, 14.14), and 2.91 days (2.55, 3.44) for Guinea, Liberia, and Sierra Leone, respectively. The contact rate, *β*(t), a measure of the force of transmission, is a function of *R*_0_(t) and *D* (Equation 6); consequently, there can be some compensation between these two parameters. Indeed, the high *R*_0_(t) for Liberia is partially offset by a higher estimated infectious period, and the low for Sierra Leone is offset by a lower estimated infectious period. In spite of its lower *R*_0_(t), Sierra Leone has the highest transmission rate, indicating the most aggressive growth, 0.34 per day, versus 0.18 per day for Guinea and 0.17 per day for Liberia on September 28, 2014. Additionally, while estimates of *R*_0_(t) have changed +5%, -56% and -46% since August 17, 2014 in Guinea, Liberia and Sierra Leone, respectively, *β*(t), a more direct measure of the outbreak growth rate, has changed +46%, -29%, +62%, respectively. These latter numbers indicate continued epidemic growth in Guinea and Sierra Leone.

Interestingly, the sum of the mean latent and infectious periods for each country is similar, 17.83 days, 15.86 days, and 15.52 days for Guinea, Liberia, and Sierra Leone, respectively. Previous estimates of the serial interval, made using case reports through September 14, 2014, gave a mean (standard deviation) of 19 (11) days, 13.1 (6.6) days, and 11.6 (5.6) days for Guinea, Liberia and Sierra Leone, respectively^2^ . These findings have the same high-to-low trend (Guinea highest, Sierra Leone lowest), are broadly consistent and are within the error bounds of our estimates of latent plus infectious periods. Note, the latent plus infectious period is not equivalent to, but rather should be longer than, serial interval. ****** ******Time from symptom onset to death is estimated as 4.70 days (3.52, 6.12), 5.86 days (4.88, 7.13), and 3.74 days (2.84, 4.78) for Guinea, Liberia, and Sierra Leone, respectively. These estimates are shorter than those obtained using a more detailed subset of total cases^2^ .******

Parameter estimates made using alternate model structures, i.e. *η* prescribed or estimated and/or *R*_0_(t) defined as in Equation 6 or estimated as a free parameter, i.e. \begin{equation*}\small{R_0(t)=R_{0Mean}}\end{equation*}, produced similar results, though *R*_0_(t) and *M* showed some sensitivity to whether *η *was prescribed or estimated through the data assimilation process (Figures S2-5). Parameter estimates made using the same core SEIRX model structure (Equations 1-6) but different initial parameter ranges revealed some sensitivity of *Z *and *D *to initial parameter range (Figures S6-9). This sensitivity was largest for *Z *estimates in Guinea and Sierra Leone.

Figure ****3 shows the ‘no change’ and ‘improved’ forecasts of cumulative incidence as generated with the optimized SEIRX following assimilation of data through September 28, 2014, as well as the forecasts generated at the 6 preceding weeks. For Liberia, the forecasts indicate that over the last 6 weeks, the ‘no change’ forecast—i.e. the optimized SEIRX model—has consistently overestimated the numbers of reported cases (Table 2). Note that with each new weekly forecast the initial conditions and parameter estimates of the model for Liberia have also shifted (Figure 2); still, the forecast cumulative incidence has been high. Even the ‘improved’ scenario forecasts with a speculative reduction in the force of transmission have overestimated reported cumulative incidence.

**No change and improved scenario forecasts of cumulative incidence for Guinea, Liberia and Sierra Leone.**
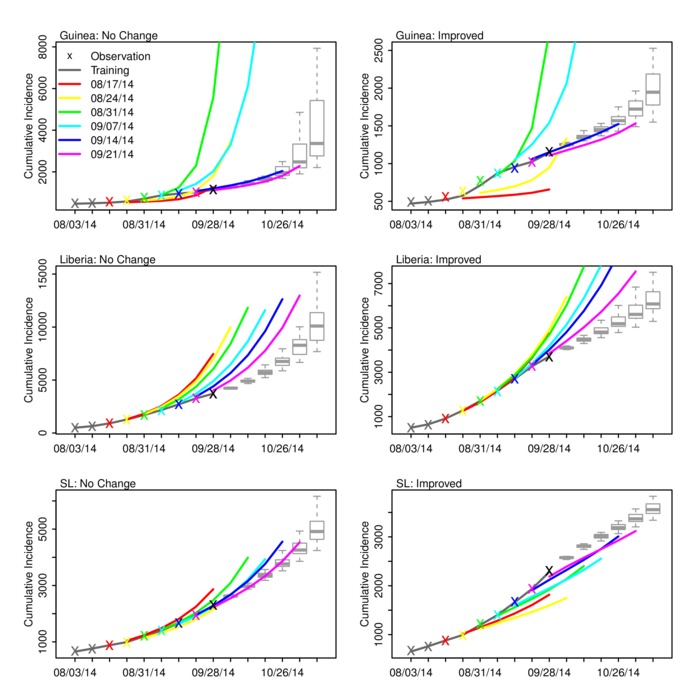
Observations (x), mean ensemble trajectory during training with observations (solid dark grey line), mean ensemble trajectory of prior weekly forecasts (colored lines), and the current forecast begun September 28, 2014 (boxes and whiskers) are shown. The colored ‘x’ shown the corresponding final observation assimilated prior to generating a given weekly ensemble forecast. The box and whiskers show the median (thick grey horizontal line), 25th and 75th percentiles (box boundaries), and 95% credible interval (whiskers).

**Table 2.** Prediction error of mean weekly incidence predictions for Liberia using the 3 forecast scenarios. Prediction error, calculated as the ratio of the difference between predicted and observed incidence to the observed incidence, in percentage (%), is shown for forecasts initiated August 17 through September 21, 2014.

		Date of Forecast					
**Observation Date**	**Scenario**	**Aug. 17**	**Aug. 24**	**Aug. 31**	**Sep. 7**	**Sep. 14**	**Sep. 21**
**Aug. 24**	**Improved**	-2.31					
	**No Change**	1.60					
	**Degraded**	5.85					
**Aug. 31**	**Improved**	-3.36	0.50				
	**No Change**	8.05	4.61				
	**Degraded**	23.39	9.16				
**Sep. 7**	**Improved**	0.05	4.05	4.27			
	**No Change**	22.14	16.53	8.56			
	**Degraded**	60.90	33.73	13.34			
**Sep. 14**	**Improved**	1.80	5.05	4.43	0.40		
	**No Change**	37.25	28.39	16.99	4.05		
	**Degraded**	118.88	71.15	34.59	8.17		
**Sep. 21**	**Improved**	9.25	11.34	9.27	2.06	0.71	
	**No Change**	62.80	49.71	33.51	12.88	4.67	
	**Degraded**	226.75	144.90	78.67	28.18	9.43	
**Sep. 28**	**Improved**	26.74	27.09	22.83	10.59	6.83	3.75
	**No Change**	109.11	89.23	64.84	32.19	19.04	8.41
	**Degraded**	430.32	292.14	171.18	72.60	39.35	14.91

In contrast, the Guinea ‘no change’ forecasts have generally been more accurate (Figure 3, Table 3). The forecasts generated following assimilation of observations through August 31 and September 7 overestimate future cumulative incidence; however, the remaining ‘no change’ forecasts match observations. Excepting the August 31 and September 7, the Guinea ‘improved’ forecasts underestimate future cumulative incidence.

**Table 3.** As in Table 2, but for Guinea.

		**Date of Forecast**					
**Observation Date**	**Scenario**	**Aug. 17**	**Aug. 24**	**Aug. 31**	**Sep. 7**	**Sep. 14**	**Sep. 21**
**Aug. 24**	Improved	-14.26					
	No Change	-13.56					
	Degraded	-12.49					
**Aug. 31**	Improved	-27.59	-19.24				
	No Change	-25.44	-17.68				
	Degraded	-19.59	-15.15				
**Sep. 7**	Improved	-34.31	-24.22	-4.29			
	No Change	-29.67	-18.21	0.50			
	Degraded	2.17	1.94	8.25			
**Sep. 14**	Improved	-37.38	-24.42	3.77	7.80		
	No Change	-27.58	-7.38	25.87	11.83		
	Degraded	136.57	135.40	111.59	17.70		
**Sep. 21**	Improved	-39.88	-21.13	22.74	13.99	2.79	
	No Change	-18.29	24.84	96.81	29.18	4.76	
	Degraded	534.67	689.61	716.57	69.62	7.58	
**Sep. 28**	Improved	-43.18	-10.89	72.91	17.17	-1.92	-4.44
	No Change	1.49	98.08	280.65	52.73	4.34	-2.78
	Degraded	1253.13	1933.43	2477.27	271.43	17.92	-0.20

For Sierra Leone, the ‘no change’ forecasts appear to have overestimated cumulative incidence 5-6 weeks in the future, but provide a better prediction than the ‘improved’ forecasts at shorter lead times (Figure 3, Table 4). For all 3 countries the ‘degraded’ forecasts overestimate future incidence and mortality (Figure S10).

**Table 4:** As in Table 2, but for Sierra Leone.

		Date of Forecast					
**Observation Date**	**Scenario**	**Aug. 17**	**Aug. 24**	**Aug. 31**	**Sep. 7**	**Sep. 14**	**Sep. 21**
**Aug. 24**	**Improved**	2.42					
	**No Change**	4.95					
	**Degraded**	7.88					
**Aug. 31**	**Improved**	-6.11	-7.93				
	**No Change**	1.51	-5.89				
	**Degraded**	12.85	-3.43				
**Sep. 7**	**Improved**	-8.70	-11.45	-0.91			
	**No Change**	6.21	-5.01	1.88			
	**Degraded**	37.24	5.05	5.36			
**Sep. 14**	**Improved**	-14.56	-18.67	-6.36	-4.13		
	**No Change**	8.79	-6.84	2.37	-1.60		
	**Degraded**	83.56	19.33	17.39	1.65		
**Sep. 21**	**Improved**	-17.22	-23.19	-9.72	-7.76	-1.56	
	**No Change**	17.68	-4.63	7.37	0.26	1.05	
	**Degraded**	197.35	59.31	52.48	13.34	4.57	
**Sep. 28**	**Improved**	-20.83	-28.98	-15.06	-14.32	-7.67	-5.00
	**No Change**	28.46	-2.92	12.11	0.56	0.34	-2.90
	**Degraded**	406.42	145.27	133.94	39.56	15.64	0.006

## Discussion

Estimation of key epidemiological parameters for the current West African Ebola outbreak is challenging, as data are limited and a full understanding of spatial-temporal complexity of Ebola transmission dynamics in the region is still lacking. Findings with our simple SEIRX model suggest that growth of Ebola in West Africa may have slowed in Liberia but continues to rise in concert with earlier prediction for Guinea and Sierra Leone. Within these countries, differences in *R*_0_(t) may manifest but can be compensated for by adjustment of the mean infectious period. As all parameters are estimated simultaneously, such compensatory action is not unexpected given the quality of observations. While the latest estimate of *R*_0_(t) for Sierra Leone was marginally below 1, the estimated contact rate, *β*(t), 0.34 per day, was much higher than for the other two countries.

Our estimates of critical Ebola epidemiological features adjust through time with each new set of observations (Figure 2). This movement, noted by others^17^ , will no doubt continue and may reflect both observational error and changes in transmission dynamics. For instance, should the virus mutate during serial passage, its epidemiological features may change in the future. More immediately, transmission dynamics may change due to shifts in population behavior, mobility, cultural practices, as information on and acceptance of the situation in affected communities changes, and as intervention measures improve or degrade.

The results presented here are specific to and limited by both the chosen model and the data used. The SEIRX model is a perfectly-mixed construct representing a highly spatially heterogeneous outbreak. This model mis-specification may produce errors in the parameter estimates and forecasts. While we have included a stochastic feature (Equation 6) to implicitly represent some possible effects of spatial heterogeneity, no spatial features are explicitly represented within each country. For instance, cases may appear to trail off due to reduced transmission in one area while growth may continue or rise in new locales^18^. These dynamics would be obscured in the aggregate country-wide observations and missed entirely by the SEIRX model. The parameter estimates made with the SEIRX model consequently represent attempts to understand the complex transmission dynamics of Ebola through the prism of a simplified simulation framework. Such projection no doubt introduces error; however, the estimates and forecasts still possess utility as the true, more complex transmission dynamics of Ebola in West Africa are under-resolved, and understanding the progression of Ebola in terms of more simplified dynamics remains informative and enables some prediction of future outcomes. As more spatially resolved surveillance records become available and understanding of how spatial connectivity within the region affects transmission dynamics improves, more spatially explicit modeling efforts will be attempted.

The observations of incidence, mortality and case fatality rates are all likely biased low. In particular, the case fatality rate data, as used here, are naively calculated from weekly cumulative mortality and incidence levels. These case fatality rates are lower than those derived from the more detailed case records housed at the WHO^2^ . We do not correct for this error here, but instead train the model to predict the biased data, including the real delay between incidence and possible death.

Given the likely unknown changing biases in the data, unknown observational error, and limited number and type of observations, the model is not as well constrained as it could be. Indeed, because of these data issues, and due to the number of degrees of freedom of the model system and system non-linearity, there appear to be limits to the constraint of the parameter estimates, so full consideration should be given to the credible intervals presented (Figure 2). In particular, as the sensitivity analysis with synthetic data indicates, *R*_0Amp _and *E* may be less well estimated.

Past ‘no change’ forecasts for Sierra Leone, as well as many for Guinea, have been in line with future observed cumulative incidence; however, for Liberia the ‘no change’ predictions have diverged from observed outcomes such that ‘improved’ scenario predictions are more accurate. A number of possible explanations for this forecast error exist, including: 1) the model is mis-specified and/or not well optimized and hence the ‘no change’ predictions consistently over-predict new case levels; 2) the data are biased low—this may be an issue if new cases and death, as reported through an increase of cumulative incidence and death on the WHO site, include delays in reporting such that some of those new cases and deaths belong to earlier weeks; such delays in reporting would slow the apparent rise in observed cases by distributing them partially in the future; as the epidemic grows exponentially such under-reporting may increase if local health care systems and infrastructure are overwhelmed; 3) the virus has changed, against evolutionary theory, and is less transmissible; 4) the virus is in areas where the effects of localized herd immunity are evident and transmission is slowing; 5) there has been an improvement in intervention and control. Which of these effects is the root of the discrepancy is not certain; however, the use of the two alternate scenarios (‘improved’ and ‘degraded’) allows some exploration of whether shifts of epidemic growth have happened rapidly. We suspect the forecast error in Liberia is principally due to data biases and, to a lesser extent, model mis-specification and improved intervention.

Because it is difficult to anticipate the scale of the global effort to control Ebola on long time horizons and how this may affect transmission dynamics, we have limited our forecasts to 6 weeks into the future. Other modeling studies have made longer term projections that simulate unimpeded outbreak growth^19^^,^ 20 . Presently, there is little indication from these modeling efforts or ours that the outbreak will extinguish any time soon without massive mobilization of intervention and control resources.

The transmission of Ebola in the current West African outbreak has also been linked to burial practices^1^ ; We have also tested an alternate model form (not shown), that explicitly accounts for transmission following mortality, which we present on our website^21^ . We will continue to generate and archive forecasts with this and other models.

## Appendix 1

### Supporting Figures

Download PDF
